# Birth asphyxia and its associated factors among newborns at a tertiary hospital: evidence from Southern Ethiopia

**DOI:** 10.4314/ahs.v23i3.17

**Published:** 2023-09

**Authors:** Mulugeta Demisse, Rahel Tadesse, Kidist Kerebeza, Yonas Alemayehu, Dawit Hoyiso, Tomas Yeheyis

**Affiliations:** School of nursing, College of medicine and health sciences, Hawassa University

**Keywords:** Birth asphyxia, neonates, neonatal intensive care unit

## Abstract

**Background:**

Globally, 45% of under-five children death occurs during the neonatal period and about 25% of all neonatal deaths are caused by birth asphyxia. In Ethiopia, in 2015, it was the first cause of neonatal deaths followed by prematurity and sepsis. The study aims to assess prevalence of Birth asphyxia and associated factors.

**Methods:**

Institution-based cross-sectional study was conducted among neonates admitted to Neonatal intensive care unit of Hawassa University Specialized comprehensive hospital from December 1 to December 30, 2020. Systematic random sampling technique was employed to select samples. Logistic regression analysis using Statistical Package for Social sciences version 24was employed.

**Results:**

The prevalence of neonatal asphyxia in this study was17.9%. Prolonged labor [AOR (Adjusted odds ration) = 2.909; (95% CI (Confidence Interval): 1.184 - 7.151)], presence of meconium [AOR= 2.137; 95% CI 1.028 - 4.683)], premature rapture of membrane [AOR = 2.459; 95% CI: 1.021 - 6.076)] and complication during labor [AOR= 3.351; 95% CI: 2.142-5.871))], were factors associated with neonatal asphyxia.

**Conclusion and Recommendations:**

Nearly two in every ten newborns faced perinatal asphyxia in the study area. Early identification of high-risk women, intervening on delay in referral, and early and vigorous management of abnormal labor and complicated labor is essential to halt the problem.

## Introduction

Birth asphyxia is oxygen deprivation that occurs around the time of birth and may be caused by several perinatal events. It is determined through utilization of a single indicator such as low Apgar (Appearance, Pulse, Grimace, Activity, and Respiration) score or delayed respiration to multiple indicators approaches focusing especially on the neurological damage related to oxygen deprivation during the birth process. Birth asphyxia shows its initial characters immediately after birth and during the neonatal period 1-3.

Globally 2.5 million children die in the first month of life which contributed to 47% of all child deaths under the age of 5-years and 54 percent of all under-five deaths occur during the neonatal period among African babies 4. Perinatal asphyxia is responsible for 23% of neonatal deaths in low-income countries. This finding underlines that perinatal asphyxia is still a burden of the world 1,2,5 However, more than two-thirds of newborns could be saved through existing maternal and child health programs though most deaths happened at home and they are invisible to the national and regional policies and programs 5. According to a World Health Organization report, perinatal asphyxia is the third leading cause of under-5 child deaths (11%) following preterm birth (17%) and pneumonia (15%) 6.

The prevalence of birth asphyxia varies across the globe and Africa contributes nearly 50% of the total; its prevalence ranges between 3.1% -56.9%. In countries with high neonatal mortality rates, this rate can be eight times higher than in those with low neonatal mortality rates 7,8,9 In Ethiopia, perinatal asphyxia is one of the leading causes of neonatal mortality, constituting 34% 8.

The effect of birth asphyxia is not limited only to death but also has a short and long-term neurodevelopment squeal, including cognitive and motor disabilities which are almost untreatable. Different studies showed that the survivors of asphyxia developed hypoxic-ischemic encephalopathy post-traumatic stress disorders, neurologic disability, low cognitive functions, and neurological sequel 9-16.

Even though birth asphyxia is the leading cause of neonatal mortality in Ethiopia, there is limited data in the southern part of the country regarding both the magnitude and determinants of birth asphyxia. Besides the trending prevalence determinants of birth asphyxia commonly noted vary across regions depending on contexts. Therefore, Identifying regional prevalence and, recognizing and managing the determinants of the problem early is of supreme importance to prevent its occurrence, reduce neonatal mortality rate, and improve neonate quality of life.

## Methods and materials

### Study area, study design, and Period

An institution-based cross-sectional study was conducted from December 1 to December 30,2020 in Hawassa University comprehensive specialized hospital (HUCSH), Hawassa, southern Ethiopia. Hawassa town is the capital city of the Sidama region and the region of Southern nation's nationalities and peoples of Ethiopia. The hospitals provide 24hour delivery, neonatal intensive care, and other medical and surgical services for Hawassa city and the surrounding areas. The Neonatal Intensive care unit (NICU) has 42-bed capacities, 4 rooms (1 preterm room, 1 term room, 1 KMC room, and 1 maternal side room.

### Population

#### Source population

The source population was all neonates admitted HUCSH in NICU from December 20/2019-November 20/2020.

#### Study population

All sampled Neonates admitted in HUCSH NICU from December 20/2019-November 20/2020.

### Eligibility criteria

All sampled records of newborns admitted to HUCSH NICU from December 20/2019-November 20/2020 were included in the study. On the other hand, records of neonates which are incomplete are excluded from the study.

### Sample Size determination and sampling procedure

The sample size was determined by using the formula for single population proportion by considering 32.8% prevalence, from the study conducted in Dilla University Referral Hospital, Southern Ethiopia, in 2019 17. Considering 95% level of confidence and 5% margin of error. According to data found from HUCSH- NICU ward, the total number of neonates admitted from December 2,2019GC to November 20,2020GC was1375, which is a total population of less than 10,000 due to this the reduction formula (nf= n_i_/1+n^i^/n) has been applied yielding a final sample size of 299.

Therefore, using a systematic random sampling technique with k=N/n which is 1375/299=5 data was collected every 5 chart intervals. The first chart was selected by using the lottery method among the first charts in respective to their chart number.

### Data collection tool and procedure

Data was collected by using a structured questionnaire through chart review. The questionnaire was adapted by reviewing different literature from various related studies. The checklist contains three parts, part one socio-demographic characteristics of the mother and the newborn (Age, sex, place of residence, educational status, occupation), part two contains an obstetric history of the mother (parity, ante partum hemorrhage, and antenatal visits, fetal presentation, mode of delivery, meconium-stained amniotic fluid, and premature rupture of membranes), and part three includes the newborn's medical history (gestational age, birth weight, comorbidity).

Data were collected using trained 3 data collectors working in HUCSH NICU. One-day training was given to the 3 data collectors and 2 experienced supervisors on the tool and the data collection procedure.

### Operational definition

Perinatal asphyxia: A newborn unable to initiate and sustain respiration, shows in an Apgar score less than 7 persistently for more than 5 min after delivery.^1^

### Data processing and Analysis

After all the questionnaires were checked visually, and categorization data was be coded, entered, and cleaned using Epi Data version 3.02 software. Errors related to inconsistency were verified using the data cleansing method. The entered data were exported and analysed with the analyses was conducted at two steps. At the first step, a bivariate logistic regression was performed (at p<0.25) for each independent and outcome of interest to identify independent predictors. Crude and adjusted odds ratios together with their corresponding 95% confidence intervals were computed to see the strength of association.

Upon the completion of the bivariable analysis, variables were selected for the multivariable analysis to control for confounding. Those variables whose bivariable test a p-value <0.25 were a candidate for multivariable model along with all variables. Once the variables were identified, multivariable analysis will begin with a model containing all of the selected variables.

Efforts was made to assess whether the necessary assumptions for the application of multiple logistic regression will be fulfilled. In this regard, the Hosmer and Lemeshow's goodness-of-fit test model coefficients was considered. A good fit as measured by Hosmer and Lemeshow's test yields a large P-value.

Finally, enter method for multivariable logistic regression model was done to determine independent predictors of the outcome variable. All tests will be two-sided and P <0.05 was considered as statistically significant. Statistical Package for Social Science (SPSS) version 24 software.

### Data quality assurance

Three days of training were given to the data collectors and supervisors. There was a brief discussion with the data collectors about the aim of the research, confidentiality of information, and content of the extraction checklist. The Collected data was checked by the principal and co-investigators for its completeness and consistency. Issues were reviewed with the supervisors and data collectors. Data were stored in the form of a file in a safe location where only the investigator has access to them, and confidentiality was secured by omitting names or any other personal information. Before data input, the data were verified once again for completeness, and following data entry, the data were cleaned by performing a basic frequency for consistency. The hard copy questionnaire will be used to check for any inconsistent data, and data analysis will then begin once these tasks have been completed.

### Ethical consideration

Ethical clearance was obtained from Hawassa University, college of medicine and health sciences Institutional Review Board. Permission was obtained from the clinical and academic director of the college of medicine and health sciences to conduct this study and to access the medical record. Confidentiality has been maintained during data collection. The study has been conducted with respect to the Declaration of Helsinki.

## Results

### Socio-demographic characteristics of the study participants

A total of 299 neonate medical records were collected from these 290 (96.9%) neonates medical records were complete and used for the study. Of the total 132 (45.4%) mothers were less than 25 years of age and 142 (48.8%) in the age group 25-35 years with the mean (SD) age was 26.89 ± 5.251. The more than half of respondents 170 (58.6%) were from urban and most of the study participants 282 (97.2%) were married. (Table 1)

**Table 1:** Socio-demographic characteristics of mothers of neonates admitted in Hawassa university comprehensive specialized hospital, Hawassa town, southern Ethiopia, 2020 (n=290)

Characteristics	Frequency	Percentages
**Age**		
<25 years	132	45.4
25-34 years	142	48.8
>35 years	16	5.5
**Place of residence**		
Urban	170	58.6
Rural	120	41.4
Marital status		
Married	282	97.2
Unmarried	8	2.8
**Family size**		
<5	184	63.2
>5	106	36.7
**Educational status**		
Primary education	253	87.2
Secondary education and above	37	12.8
**Occupation**		
Housewife	280	96.6
Merchant	10	3.4

### Obstetric history of the respondents

From the reviewed document the majority 193 (66.6%) of them had more than two pregnancies and 97 (33.4%) of them were primigravida. Two hundred eighty-four (97.9%) of them had ANC follow-up for their current pregnancy, while the remaining 6 (2.1%) of them do not have ANC follow-up. (Table 2)

**Table 2:** Basic obstetric history of mothers who delivered in Hawassa university comprehensive specialized hospital, Hawassa town, southern Ethiopia, 2020 (n=290)

Characteristics	Frequency	Percentages
**Parity**		
Primigravida	97	33.4
Multigravida	193	66.6
**ANC follow up in this pregnancy**		
Yes	284	97.9
No	6	2.1
**Number of ANC follow up**		
<4 times	16	5.5
> 4 times	274	94.5
**Gestational age at birth**		
<37 weeks	68	23.4
≥37weeks	222	76.6
**Presence of comorbid medical illness**		
Yes	46	15.9
No	242	83.4
**History of adverse birth outcomes before this pregnancy**		
Yes	35	12.1
No	255	87.9s

### Prevalence of birth asphyxia

**Fig. 1:**
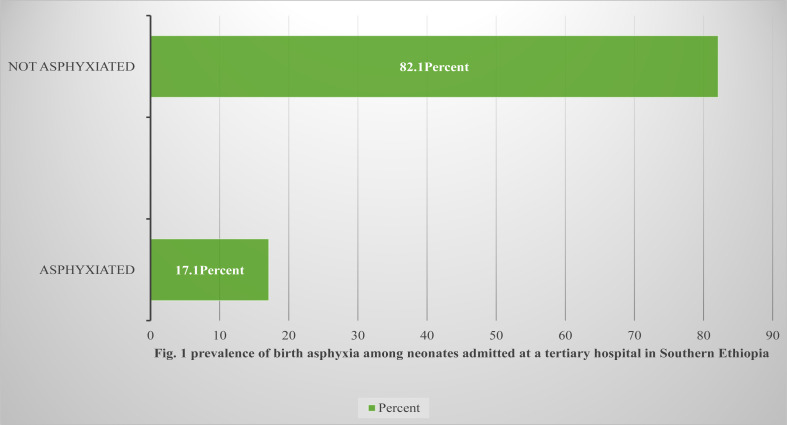
prevalence of birth asphyxia among neonates admitted at a tertiary hospital in Southern Ethiopia

Among the total of 290 newborns assessed in study 52 (17.9%) had birth asphyxia and the rest 238 (82.1%) didn't experience birth asphyxia. (Figure 1)

### Factors associated with birth asphyxia

Binary logistic regression showed that complication/ danger signs during pregnancy, a medical problem during pregnancy, prolonged duration of labor, meconium-stained amniotic fluid, and Presence of complication during labor, and malpresentation and premature rupture of membrane/PROM were significantly associated.

After that, the variables which showed an association in the bivariate model entered into the multivariate regression analysis model. The multivariable logistic regression analysis revealed that prolonged duration of labor increases the odds of developing birth asphyxia by 2.9 times [AOR= 2.909; (95% CI:1.184 – 7.151)]. Women who have meconium-stained amniotic fluid were2.1 times more likely to deliver newborns with birth asphyxia than those who have clear amniotic fluid [AOR= 2.137; (95% CI:1.028 - 4.683)]. On the other hand, those women who had a complication during labor were 3.3 times more likely to birth asphyxiated newborns than women with who have no complication during labor[AOR= 3.351; (95% CI:2.142 - 5.871)]. Women those who have PROM were2.4 times more likely to deliver newborn with birth asphyxia than those who had normal labor [AOR= 2.459; (95% CI:1.021 - 6.076)”.

## Discussion

In this study, the prevalence of prenatal asphyxia was found to be 17.9%, this prevalence is higher than systematic review and meta-analysis conducted on the Prevalence of perinatal asphyxia in East and Central Africa: which is 15.9%^11^. But the result in this study is comparable with the prevalence rate was seen in the Ethiopian meta-analysis study conducted among 15 studies conducted in Ethiopia, 21%^13^. Moreover, it is also comparable with the studies done in other African countries like Gusau, Nigeria (21.1%)^14^, and Dares Salaam, Tanzania (21.1%) 15. But it was lower than the study conducted in Dilla, Southern Ethiopia, which was 32.8%^17^. This high rate of variation could be due to differences in the methodology, the use of different definitions of birth asphyxia in different settings. More the lower asphyxia prevalence in the study compared to other studies conducted in Ethiopia might be because this study is done in a specialized hospital but others have been conducted in regional and general hospitals which creates a difference in the availability of resources and trained health workers, in addition, socio-cultural disparity or difference in the study participants' economic status have played its part.

The current study shows that prolonged duration of labor increases the odds of developing birth asphyxia by 2.9 times [AOR= 2.909; (95% CI:1.184 - 7.151)]. This is consistent with studies conducted in different hospitals in Ethiopia including general hospitals in Tigray, Dessie, and Dire Dawa 13, 22,23 and in other African countries like Bayero University Kano, Nigeria^18^ and Cameroon^24^. The reason could be delayed labor which might be causing the fetus to be involved in labor for a long time that in turn, distress in the newborn increases the newborn's probability of drinking, and aspiration of amniotic fluid carries a higher risk of birth trauma and asphyxia. In addition, as labor prolongs it needs caution and rigorous interventions from the professionals, which can be missed by professionals and may result in the asphyxia of the newborn.

Women who have meconium-stained amniotic fluid were 2.1 times more likely to birth asphyxia than those who have clear amniotic fluid [AOR= 2.137; (95% CI:1.028 - 4.683)]. This finding was consistent with a conducted in the general hospitals of Tigray^13^ and also studies conducted in developing countries, and low and middle-income countries 20,21. Meconium-stained amniotic fluid is often associated with fetal hypoxia. Asphyxia from meconium aspiration results from the combination of airway obstruction, pulmonary hypertension, epithelial injury, surfactant inactivation, and inflammation. Moreover, meconium discharge in the amniotic fluid and in turn gasping and aspiration of meconium-stained amniotic fluid changes muscular elasticity of pulmonary blood vessels of the fetus^25^.

Those women who had a complication during labor were 3.3 times more likely have to birth asphyxia [AOR= 3.351; (95% CI: 2.142 - 5.871)] than women with those who have no complication during labor. This finding was consistent with other Ethiopian studies 17,26. A Cameroonian study^24^ also showed a similar finding. There can be different complications during labor. Some of the complications during labor like umbilical cord prolapse where the umbilical cord leaves the cervix before the baby which results in suffocation and aspiration of amniotic fluid in the newborn. Similarly, anemia can result in birth asphyxia. In a mother and newborn with anemia, the red blood cells don't carry enough oxygen thus the newborn will be subjected to oxygen deficiency.

This study shows that women who have premature membrane rupture were 2.4 times more likely to have a newborn with birth asphyxia c than those who normal labor [AOR= 2.459; (95% CI: 1.021 - 6.076)]. A consistent finding was obtained from studies at Cameroon^24^, Uganda^27^, and Al-Diwaniya teaching hospital in Brazil^28^. This consistency can be justified by the fact that when fetal membranes rupture prematurely, a spontaneous gush of amniotic fluid happens along with the prolapse of the umbilical cord which is in turn accompanied by cord compression and subsequent asphyxia at birth. Moreover, premature rupture of membranes, if prolonged, often facilitates feto-maternal systemic infections which are usually ensured by subsequent neonatal asphyxia.

## Conclusions

Unacceptably high proportion, nearly two in every ten, of newborns faced perinatal asphyxia in the study area. Prolonged labor, presence of meconium, premature rapture of membrane and complication during labor were positive predictors of neonatal asphyxia. Early identification of high-risk women, intervening on delay in referral, and early, cautious and vigorous management of abnormal labor and complicated labor is essential to minimize the risk of perinatal asphyxia in newborns.

## Figures and Tables

**Table 3:** Results of Bivariate and Multivariate analysis on factors associated with birth asphyxia among mothers delivered in Hawassa university comprehensive specialized hospital, Hawassa town, southern Ethiopia, 2020

Variables	No Prenatal asphyxia	Prenatal asphyxia	AOR (95% C.I.)	P. value
**Presence of pregnancy-related complication**				
Yes	18	10	0.330 (0.139-1.782)	0.012
No	219	42	1	
**Presence of medical problems during pregnancy**				
Yes	31	15	0.669 (0.280-1.594)	0.364
No	205	37	1	
**Pronged labor**				
Yes	23	11	2.909 (1.184 - 7.151)	7.151**
No	215	41	1	
**Meconium-stained amniotic fluid**				
Yes	203	46	2.137 (1.028 - 4.683)	0.015*
No	35	6	1	
**Complication during labor**				
Yes	24	17	3.351 (2.142 - 5.871)	0.024*
No	214	35	1	
**Malpresentation**				
Yes	39	19	0.354 (0.189-0763)	0.057
No	198	33	1	
**PROM**				
Yes	29	12	2.459 (1.021 - 6.076)	0.013*
No	209	40	1	

**Complications during labour:** placenta Previa, placenta abraptio, bleeding and fetal distress

## Data Availability

The datasets used and/or analysed during the current study are available upon request from the corresponding author and Co-authors.

## Abbreviations

APGAR: Appearance, pulse, grimace, activity, and respiration
EFY: Ethiopian fiscal year
HIE: Hypoxic ischemic encephalopathy
NICU: Neonatal intensive care unit
PNA: Peri-natal asphyxia
RDS: Respiratory distress syndrome
SPSS: Statistical package for social sciences
WHO: World health organization
LMICs: Low and middle-income countries
NMR: Neonatal mortality rate
